# BPRMeth: a flexible Bioconductor package for modelling methylation profiles

**DOI:** 10.1093/bioinformatics/bty129

**Published:** 2018-03-07

**Authors:** Chantriolnt-Andreas Kapourani, Guido Sanguinetti

**Affiliations:** 1 School of Informatics, University of Edinburgh, Edinburgh, UK; 2 Synthetic and Systems Biology, University of Edinburgh, Edinburgh, UK

## Abstract

**Motivation:**

High-throughput measurements of DNA methylation are increasingly becoming a mainstay of biomedical investigations. While the methylation status of individual cytosines can sometimes be informative, several recent papers have shown that the functional role of DNA methylation is better captured by a quantitative analysis of the spatial variation of methylation across a genomic region.

**Results:**

Here, we present BPRMeth, a Bioconductor package that quantifies methylation profiles by generalized linear model regression. The original implementation has been enhanced in two important ways: we introduced a fast, variational inference approach that enables the quantification of Bayesian posterior confidence measures on the model, and we adapted the method to use several observation models, making it suitable for a diverse range of platforms including single-cell analyses and methylation arrays.

**Availability and implementation:**

http://bioconductor.org/packages/BPRMeth

**Supplementary information:**

Supplementary data are available at *Bioinformatics* online.

DNA methylation is probably the best studied epigenomic mark, due to its well established heritability and widespread association with diseases. Yet its role in gene regulation and the molecular mechanisms underpinning its association with diseases are still imperfectly understood. While methylation of CpG islands is widely recognized as a prominent gene silencing mechanism, the importance of DNA methylation in different genomic regions, such as gene bodies, remains largely mysterious.

While many early studies concentrated on methylation levels at single CpGs, or average levels across regions, recent years have gradually seen a shift in perspective towards considering spatial patterns of DNA methylation in more detail. Methods considering spatial methylation patterns have been successful in more effectively predicting changes in gene expression (Vanderkraats *et al.*, 2013) and in developing powerful statistical tests for differential methylation (Mayo *et al.*, 2015).

Recently, we proposed a method to quantify explicit features of methylation profiles, in a way that would make it easier to formally use such profiles in downstream modelling efforts (Kapourani and Sanguinetti, 2016). Mathematically, the approach is based on a basis function generalized linear model. The basic idea is as follows: the methylation profile associated with a genomic region *D* is defined as a (latent) function *f*: *D* → (0, 1), which takes as input the genomic coordinate along the region and returns the propensity for that locus to be methylated. To enforce spatial smoothness and obtain a compact representation for this function in terms of interpretable features, we represent the profile function as a linear combination of basis functions

(1) $$f(x)=\Phi \left(w^{T}h(x)\right),$$

where *h*(*x*) are the basis functions, and Φ is a probit transformation needed in order to map the function output to the (0, 1) interval. The latent function is observed at specific loci through a noise model that encapsulates the experimental technology. The optimal weight parameters **w** can be recovered by maximum likelihood estimation, providing a set of quantitative features that can be used in downstream models such as prediction of gene expression and clustering of regions according to their methylation profiles.

The original paper (Kapourani and Sanguinetti, 2016) provides a full derivation of the mathematical model and demonstrates on a number of datasets the usefulness of the methylation profile approach. In this application note, we describe the features of the software supporting this model. Importantly, with respect to the original implementation, the scope and flexibility of the software have been extended considerably. The major features of this new implementation are as follows:

- The R implementation is now supported by full documentation and examples, available in the Bioconductor package BPRMeth.
- BPRMeth supports single cell methylation data, using a Bernoulli likelihood (see Section 1.1 in Supplementary Material).
- BPRMeth supports data measured by methylation array platforms achieved by using a Beta likelihood (Siegmund, 2011) (see Section 1.2 in Supplementary Material).
- BPRMeth now supports approximate Bayesian estimation via mean-field variational inference (Blei *et al.*, 2017). This enables to perform model selection and quantify uncertainty in all model quantities *a posteriori* (see Section 2 in Supplementary Material).
- BPRMeth supports Fourier basis functions, as well as radial and polynomial basis functions, which might be useful for analysing data with expected periodicity (e.g. nucleosome positioning).

We notice that, while the main purpose of BPRMeth is to provide a flexible tool for methylation data, the approach is in principle deployable to other measurements with a similar structure, and indeed the method was already used for single-cell chromatin accessibility data in Clark *et al.* (2018).

The operational characteristics of the software are as follows: A methylation and annotation file are given as input to create genomic regions of pre-specified length. The annotation file might contain arbitrary genomic contexts, e.g. promoters or enhancers, Next, a basis object is required to transform the input methylation data, e.g. the create_rbf_object will produce an RBF object. The infer_profiles_vb (variational inference) or infer_profiles_mle (maximum likelihood estimation) functions are used to infer the latent methylation profiles. Equivalently, the cluster_profiles_vb or cluster_profiles_mle functions are used to cluster genomic regions. The execution times of the algorithm, using 10 kb windows on 5000 promoters takes around 5 min for inferring profiles and 20 min for clustering.

The output of the algorithm can then be used for downstream analyses, such as predicting gene expression (using the predict_expr function) or quantifying levels of accessibility heterogeneity across cells, see Clark *et al.* (2018). To visualize the results, the objects produced from the model are given as input to plot_infer_profiles or plot_cluster_profiles. An example of the graphical output of the software is given in Figure 1.

**Fig. 1.**
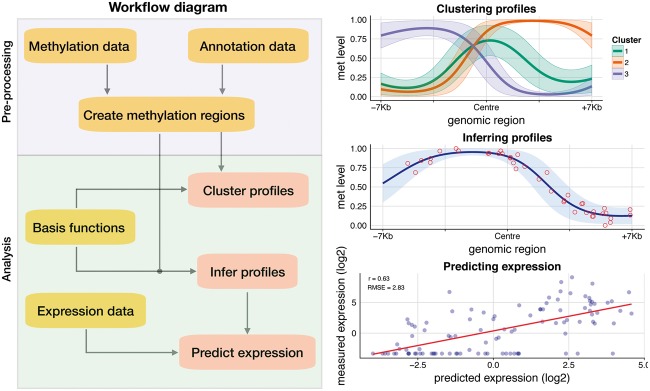
Analysis schematic workflow (left) with example output graphs (right)

In conclusion, BPRMeth provides a flexible environment to analyse and model spatial patterns in DNA methylation and similarly structured data from a variety of experimental platforms. Given the continuing popularity of methylome assays, and their rapid expansion in a clinical setting, we expect BPRMeth to become a widespread tool in the high-throughput bioinformatics workbench.

## Funding

This work was supported in part by the EPSRC Centre for Doctoral Training in Data Science [grant EP/L016427/1] and the University of Edinburgh to C.A.K.

*Conflict of Interest*: none declared.

## Supplementary Material

Supplementary Data
