# Supplementary material for: BPRMeth: a flexible Bioconductor package for modelling methylation profiles
Source: Bioinformatics. 2018 Mar 7;34(14):2485–6. doi: 10.1093/bioinformatics/bty129 (PMC6041802; doi:10.1093/bioinformatics/bty129)
Supplement: Supplementary Data [file bty129_cak_gs-2018_bprmeth_supp.pdf]

Online supplementary information for the paper

BPRMeth: a flexible Bioconductor package for modelling methylation profiles

Chantriolnt-Andreas Kapourani and Guido Sanguinetti

1 Maximum Likelihood Estimation (MLE)

For all three observation models considered in the paper (i.e. Binomial, Bernoulli and Beta regression models) there is no closed form solution for the maximum likelihood estimate (MLE) due to the presence of the probit transformation, hence, we perform numerical optimization using the Conjugate Gradients[1] method (Hestenes and Stiefel, 1952). The Conjugate Gradients method is a first order numerical optimization algorithm, thus, we need to derive both the likelihood and the gradient for each observation model. Similarly to the derivation for the Binomial model (Kapourani and Sanguinetti, 2016), we can obtain the MLE for the Bernoulli and Beta likelihood models.

1.1 Bernoulli observation model

To infer methylation profiles for single cell methylation data (Clark *et al.*, 2018; Smallwood *et al.*, 2014), we use a Bernoulli observation model. Briefly, assume that for a genomic region D, we have I independent CpGs $y_i \in \{0, 1\}$, then the log-likelihood of the Bernoulli probit regression model is given by

$$\begin{aligned} \ln p(\mathbf{y}|\mathbf{x}, \mathbf{w}) &= \ln \left[\prod_{i=1}^{I} \mathcal{B}ernoulli\big(y_i | \Phi(\mathbf{w}^T \mathbf{h}(x_i))\big) \right] \\ &= \sum_{i=1}^{I} \ln \mathcal{B}ernoulli\big(y_i | \Phi(\mathbf{w}^T \mathbf{x}_i)\big), \end{aligned} \tag{1}$$

where $\mathbf{x}_i \equiv \mathbf{h}(x_i)$ denotes the the basis function evaluations at CpG site x_i, $\mathbf{w} \in \mathbb{R}^D$ represent the regression coefficients, and Φ is the inverse probit function (Gaussian cumulative distribution function) needed in order to map the function output to the $(0, 1)$ interval. The gradient of Eq. 1 with respect to model parameters $\mathbf{w}$ is given by

$$\begin{aligned} \nabla_{\mathbf{w}} \ln p(\mathbf{y}|\mathbf{x}, \mathbf{w}) &= \sum_{i=1}^{I} \nabla_{\mathbf{w}} \left[\ln \mathcal{B}ernoulli\Big(y_i | \Phi(\mathbf{w}^T \mathbf{x}_i)\Big) \right] \\ &= \sum_{i=1}^{I} \left[\Big(\Phi(\mathbf{w}^T \mathbf{x}_i)^{-1} \big(1 - \Phi(\mathbf{w}^T \mathbf{x}_i)\big)^{-1} \Big) \phi(\mathbf{w}^T \mathbf{x}_i) \mathbf{x}_i \right], \end{aligned} \tag{2}$$

where $\phi(\cdot)$ is the probability density function for the standard normal distribution $\mathcal{N}(0, 1)$.

[1] Conjugate Gradients method can be replaced by any numerical optimisation approach, e.g. BFGS or Gradient descent.

1.2 Beta observation model

To infer methylation profiles for array methylation data and generally any continuous observations that lie in (0, 1) interval, we use a Beta observation model which has been successfully used for DNA methylation arrays in several earlier publications, see e.g. Siegmund (2011).

We use a different parameterisation of the beta regression model (Ferrari and Cribari-Neto, 2004) in terms of a mean μ and a precision parameter γ. The Beta density then takes the form

$$\mathcal{B}eta(y|\mu,\gamma) = \frac{\Gamma(\gamma)}{\Gamma(\mu\gamma)\Gamma((1-\mu)\gamma)} y^{\mu\gamma-1}(1-y)^{(1-\mu)\gamma-1}, \tag{3}$$

with $0 < \mu < 1$ and $\gamma > 0$ and $\Gamma(\cdot)$ is the *Gamma* function.

Assume that for a genomic region D, we have I independent CpGs $y_i \in (0,1)$, then the log-likelihood of the Beta probit regression model becomes

$$\begin{aligned} \ln p(\mathbf{y}|\mathbf{x},\mathbf{w}) &= \ln\left[\prod_{i=1}^{I}\mathcal{B}eta\big(y_i|\Phi(\mathbf{w}^T\mathbf{x}_i),\gamma\big)\right] \\ &= \sum_{i=1}^{I}\Bigg[\ln\Gamma(\gamma) - \ln\Gamma\left(\Phi(\mathbf{w}^T\mathbf{x}_i)\gamma\right) - \ln\Gamma\left((1-\Phi(\mathbf{w}^T\mathbf{x}_i))\gamma\right) \\ &\qquad + \left(\Phi(\mathbf{w}^T\mathbf{x}_i)\gamma - 1\right)\ln y_i + \Big(\left(1-\Phi(\mathbf{w}^T\mathbf{x}_i)\right)\gamma - 1\Big)\ln[1-y_i]\Bigg]. \end{aligned} \tag{4}$$

The gradient of Eq. 4 with respect to model parameters $\mathbf{w}$ is given by

$$\begin{aligned} \nabla_{\mathbf{w}} \ln p(\mathbf{y}|\mathbf{x},\mathbf{w}) &= \sum_{i=1}^{I}\nabla_{\mathbf{w}}\left[\ln\mathcal{B}eta\Big(y_i|\Phi(\mathbf{w}^T\mathbf{x}_i),\gamma\Big)\right] \\ &= \sum_{i=1}^{I}\gamma\phi(\mathbf{w}^T\mathbf{x}_i)\Big[\ln y_i - \ln[1-y_i] - \psi\left(\Phi(\mathbf{w}^T\mathbf{x}_i)\gamma\right) + \psi\Big(\left(1-\Phi(\mathbf{w}^T\mathbf{x}_i)\right)\gamma\Big)\mathbf{x}_i\Big], \end{aligned} \tag{5}$$

where $\psi(\cdot)$ is the *digamma* function. Note that in this formulation the precision parameter γ is the same across observations. Also, the current implementation does not allow to infer the precision parameters γ, which are assumed to be fixed. We leave the extension of jointly optimising over the mean μ and precision γ as future work.

2 Variational Bayes

In mean-field variational inference (Blei *et al.*, 2017) the intractable posterior probability distribution of the latent variables $p(\boldsymbol{\theta}|\mathbf{X})$ is approximated by a factorized distribution $q(\boldsymbol{\theta}) = \prod_i q_i(\boldsymbol{\theta}_i)$, where $\boldsymbol{\theta}$ denotes the latent variables and $\mathbf{X}$ the observed variables. Then we search over the space of approximating distributions to find the distribution with the minimum Kullback-Leibler ($\mathcal{KL}$) divergence with the actual posterior

$$\mathcal{KL}(q(\boldsymbol{\theta})\ ||\ p(\boldsymbol{\theta}|\mathbf{X})) = -\int q(\boldsymbol{\theta})\ln\frac{p(\boldsymbol{\theta}|\mathbf{X})}{q(\boldsymbol{\theta})}d\boldsymbol{\theta}. \tag{6}$$

The $\mathcal{KL}$ divergence can then be minimised by performing a free form minimisation over the $q_i(\boldsymbol{\theta}_i)$ leading to the following update equation

$$q_i(\boldsymbol{\theta}_i) = \frac{\exp\left\langle \ln p(\mathbf{X},\boldsymbol{\theta})\right\rangle_{q_{j\neq i}}}{\int\exp\left\langle \ln p(\mathbf{X},\boldsymbol{\theta})\right\rangle_{q_{j\neq i}}d\boldsymbol{\theta}_i} \tag{7}$$

where $\langle\cdot\rangle_{q_{j\neq i}}$ denotes an expectation with respect to the distributions $q_j(\boldsymbol{\theta}_j)$ for all $j \neq i$.

2.1 Inferring methylation profiles

2.1.1 Bernoulli observation model

To account for the inherent noise measurements and the limited CpG coverage, we also reformulate the model in a Bayesian framework. The Bayesian probit regression model for a single CpG site then becomes

$$\begin{aligned} y_i &\sim \mathcal{B}ernoulli\left(y_i|\Phi\left(\mathbf{w}^T\mathbf{x}_i\right)\right), \\ \mathbf{w} &\sim p(\mathbf{w}|\mathcal{H}), \end{aligned} \tag{8}$$

Performing inference for this model in the Bayesian framework is complicated by the fact that no conjugate prior $p(\mathbf{w}|\mathcal{H})$ exists for the parameters of the probit regression model. The model can be made amenable to Bayesian estimation thanks to a data augmentation strategy originally proposed by Albert and Chib (1993). This strategy consists of introducing an additional auxiliary latent variable z_i, which has a Gaussian distribution conditioned on the input $\mathbf{w}^T\mathbf{x}_i$. The augmented model has the hierarchical structure shown in Fig. 1, where y_i is now deterministic conditional on the sign of the latent variable z_i. Now we introduce a conjugate Gaussian prior over the parameters $\mathbf{w} \sim \mathcal{N}(\mathbf{w}|\mathbf{0}, \tau^{-1}\mathbf{I})$, where the hyper-parameter τ controlling the precision of the Gaussian prior is assumed to follow a Gamma distribution. This reduces the necessary conditional distributions to a tractable form as either Gaussian, Gamma or one-dimensional truncated Gaussian distributions.

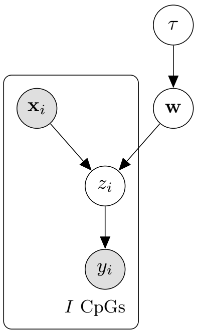

$$\begin{aligned} \tau &\sim Gamma(\tau|\alpha_0, \beta_0) \\ \mathbf{w}|\tau &\sim \mathcal{N}(\mathbf{w}|\mathbf{0}, \tau^{-1}\mathbf{I}) \\ z_i|\mathbf{w}, \mathbf{x}_i &\sim \mathcal{N}(z_i|\mathbf{w}^T\mathbf{x}_i, 1) \\ y_i|z_i &= \begin{cases} 1 & \text{if } z_i > 0 \\ 0 & \text{if } z_i \leq 0 \end{cases} \end{aligned}$$

Figure 1: Probabilistic graphical representation of the Bayesian probit regression model.

Thus, the the joint distribution over all the variables is given by

$$\begin{aligned} p(\mathbf{y}, \mathbf{z}, \mathbf{w}, \tau|\mathbf{X}) &= p(\mathbf{y}|\mathbf{z})p(\mathbf{z}|\mathbf{w}, \mathbf{X})p(\mathbf{w}|\tau)p(\tau) \\ &= \left\{\prod_{i=1}^{I} p(y_i|z_i)p(z_i|\mathbf{x}_i, \mathbf{w})\right\} p(\mathbf{w}|\tau)p(\tau). \end{aligned} \tag{9}$$

To apply the variational inference machinery, we take the variational posterior distribution to factorise over the latent variables

$$q(\mathbf{z}, \mathbf{w}, \tau) = q(\mathbf{z})q(\mathbf{w})q(\tau) \simeq p(\mathbf{z}, \mathbf{w}, \tau|\mathbf{y}, \mathbf{X}). \tag{10}$$

Applying Eq. (7) to our model, we obtain the following solutions for the optimised factors of the variational posterior[2]

$$\begin{aligned} q(\mathbf{z}) &= \prod_{i=1}^{I} \begin{cases} \mathcal{TN}_+\left(z_i|\left\langle\mathbf{w}^T\mathbf{x}_i\right\rangle_{q(\mathbf{w})}, 1\right) & \text{if } y_i = 1 \\ \mathcal{TN}_-\left(z_i|\left\langle\mathbf{w}^T\mathbf{x}_i\right\rangle_{q(\mathbf{w})}, 1\right) & \text{if } y_i = 0 \end{cases} \\ q(\tau) &= \mathcal{G}amma\left(\tau|\alpha_0 + \frac{D}{2}, \beta_0 + \frac{1}{2}\left\langle\mathbf{w}^T\mathbf{w}\right\rangle_{q(\mathbf{w})}\right), \\ q(\mathbf{w}) &= \mathcal{N}(\mathbf{w}|\mathbf{m}, \mathbf{S}), \end{aligned} \tag{11}$$

[2]Detailed mathematical derivations can be found in http://rpubs.com/cakapourani/variational-bayes-bpr

where

$$\mathbf{m} = \mathbf{S}\mathbf{X}^T \ \langle \mathbf{z} \rangle_{q(\mathbf{z})},$$
$$\mathbf{S} = \left(\langle \tau \rangle_{q(\tau)} \mathbf{I} + \mathbf{X}^T\mathbf{X} \right)^{-1},$$

and $\mathcal{TN}_+(\cdot)$ denotes the normal distribution truncated on the left tail to zero to contain only positive values, and $\mathcal{TN}_-(\cdot)$ denotes the normal distribution truncated on the right tail to zero to contain only negative values. The variational lower bound (i.e. evidence lower bound) which we are optimising over is given by

$$\begin{aligned}
\mathcal{L}(q) &= \int\int\int q(\mathbf{z}, \mathbf{w}, \tau) \ln \left| \frac{p(\mathbf{y}, \mathbf{z}, \mathbf{w}, \tau | \mathbf{X})}{q(\mathbf{z}, \mathbf{w}, \tau)} \right| d\mathbf{z}\, d\mathbf{w}\, d\tau \\
&= \langle \ln p(\mathbf{y}, \mathbf{z}, \mathbf{w}, \tau | \mathbf{X}) \rangle_{q(\mathbf{z}, \mathbf{w}, \tau)} - \langle \ln q(\mathbf{z}, \mathbf{w}, \tau) \rangle_{q(\mathbf{z}, \mathbf{w}, \tau)} \\
&= \langle \ln p(\mathbf{y}|\mathbf{z}) \rangle_{q(\mathbf{z})} + \langle \ln p(\mathbf{z}|\mathbf{X}, \mathbf{w}) \rangle_{q(\mathbf{z}, \mathbf{w})} + \langle \ln p(\mathbf{w}|\tau) \rangle_{q(\mathbf{w}, \tau)} + \langle \ln p(\tau) \rangle_{q(\tau)} \\
&\quad - \langle \ln q(\mathbf{z}) \rangle_{q(\mathbf{z})} - \langle \ln q(\mathbf{w}) \rangle_{q(\mathbf{w})} - \langle \ln q(\tau) \rangle_{q(\tau)}.
\end{aligned} \tag{12}$$

The predictive distribution over y_*, given a new input $\mathbf{x}_*$, is evaluated using the variational posterior for the parameters

$$\begin{aligned}
p(y_*|\mathbf{x}_*, \mathbf{X}, \mathbf{y}) &= \int\int\int p(y_*, z, \mathbf{w}, \tau | \mathbf{x}_*, \mathbf{X}, \mathbf{y})\, d\tau\, d\mathbf{w}\, dz \\
&\simeq \int\int\int p(y_*|z)\, p(z|\mathbf{w}, \mathbf{x}_*) q(\mathbf{w}) q(\tau)\, d\tau\, d\mathbf{w}\, dz \\
&= \int \mathbf{1}(z > 0)^{y_*} \mathbf{1}(z \leq 0)^{(1-y_*)}\, \mathcal{N}(z|\mathbf{m}^T\mathbf{x}_*, 1 + \mathbf{x}_*^T\mathbf{S}\mathbf{x}_*)\, dz \\
&= \mathcal{B}ernoulli\left(y_* \middle| \Phi\left(\frac{\mathbf{m}^T\mathbf{x}_*}{(1 + \mathbf{x}_*^T\mathbf{S}\mathbf{x}_*)^{1/2}} \right) \right).
\end{aligned} \tag{13}$$

2.1.2 Binomial observation model

For the Binomial observation model, we can recast it as a Bernoulli observation model with additional observations. Assume that we have I independent observations $y_i = (m_i, T_i)$ that follow a Binomial distribution

$$m_i \sim \mathcal{B}inomial(m_i|T_i, \gamma_i), \tag{14}$$

where T_i is the total number of trials for the ith observation, m_i denotes the number of successes and again the success probability γ_i is related to a vector of covariates $\mathbf{x}_i \in \mathbb{R}^D$. We can think of each of the m_i as the total number of successes of T_i independent Bernoulli experiments with outcomes $y_{i1}^*, ..., y_{iT_i}^*$, where now each y_{it}^* follows a Bernoulli distribution. It is simple to reconstruct the binary outcomes y_{it}^* from the Binomial observations T_i using the following formula,

$$y_{it}^* = \begin{cases} 1 & \text{if } 1 \leq t \leq m_i \\ 0 & \text{if } m_i < t \leq T_i \end{cases} \tag{15}$$

Using this approach of extending our observation matrix to binary outcomes, now we can use the variational implementation for the Bernoulli regression observation model we derived above to perform inference for Binomial data.

2.1.3 Beta observation model

In the case of Beta observation model, the data augmentation strategy cannot be applied hence we cannot introduce a conjugate prior over the coefficients that would lead to tractable form conditional distributions. Due to these difficulties, we have left the variational Bayes implementation of the Beta observation model as an interesting topic for future work.

2.2 Clustering methylation profiles

Imagine that each of our observations comprises of a different basis function regression models and our goal is to cluster together regression models with similar patterns, e.g. cluster genomic regions with similar methylation patterns or for a given genomic region cluster cells based on their methylation profiles. An concrete example is shown in the next section.

2.2.1 Bernoulli observation model

Assume that we have $N (n = 1, ..., N)$ observations and each of our observations is generated from a corresponding latent variable $\mathbf{c}_n$ comprising a 1-of-K binary vector with elements c_{nk} for $k = 1, ..., K$. The conditional distribution of $\mathbf{C}$, given the mixing proportions $\boldsymbol{\pi}$, is given by

$$p(\mathbf{C}|\boldsymbol{\pi}) = \prod_{n=1}^{N} \prod_{k=1}^{K} \pi_k^{c_{nk}}, \tag{16}$$

The latent variables $\mathbf{C}$ will generate our latent observations $\mathbf{Z} \in \mathbb{R}^{N \times I_n}$, which in turn will generate our binary observations $\mathbf{Y} \in \mathbb{R}^{N \times I_n}$ depending on the sign of $\mathbf{Z}$ as explained above. The conditional distribution of the data $(\mathbf{Z}, \mathbf{Y})$, given the latent variables $\mathbf{C}$ and the component parameters $\mathbf{w}$ is

$$p(\mathbf{Y}, \mathbf{Z}|\mathbf{C}, \mathbf{w}, \mathbf{X}) = \prod_{n=1}^{N} \prod_{k=1}^{K} \left[p(\mathbf{y}_n|\mathbf{z}_n)\ p(\mathbf{z}_n|\mathbf{w}_k, \mathbf{X}_n)\right]^{c_{nk}}. \tag{17}$$

To complete the model we introduce priors over the parameters. We choose a Dirichlet distribution over the mixing proportions $\boldsymbol{\pi} \sim \mathcal{Dir}(\boldsymbol{\pi}|\boldsymbol{\delta}_0)$, an independent Gaussian prior over the coefficients for each cluster $\mathbf{w}_k \sim \mathcal{N}(\mathbf{w}_k|\mathbf{0}, \tau_k^{-1}\mathbf{I})$ and finally a Gamma prior for the precision parameter $\tau_k \sim \mathcal{Gamma}(\tau_k|\alpha_0, \beta_0)$.

Having defined our model, we can now write the joint distribution over the observed and latent variables

$$p(\mathbf{Y}, \mathbf{Z}, \mathbf{C}, \mathbf{w}, \boldsymbol{\pi}, \boldsymbol{\tau}|\mathbf{X}) = p(\mathbf{Y}|\mathbf{Z})\ p(\mathbf{Z}|\mathbf{C}, \mathbf{w}, \mathbf{X})\ p(\mathbf{C}|\boldsymbol{\pi})\ p(\boldsymbol{\pi})\ p(\mathbf{w}|\boldsymbol{\tau})\ p(\boldsymbol{\tau}), \tag{18}$$

For out probabilistic model, the approximating distribution factorises over the latent variables as follows

$$q(\mathbf{Z}, \mathbf{C}, \mathbf{w}, \boldsymbol{\pi}, \boldsymbol{\tau}) = q(\mathbf{Z})\ q(\mathbf{C})\ q(\mathbf{w})\ q(\boldsymbol{\pi})\ q(\boldsymbol{\tau}). \tag{19}$$

Applying Eq. (7) to our model, we obtain the following solutions for the optimised factors of the variational posterior[3]

$$\begin{aligned}
q(\mathbf{C}) &= \prod_{n=1}^{N} \prod_{k=1}^{K} r_{nk}^{c_{nk}} \qquad \text{where} \quad r_{nk} = \frac{\rho_{nk}}{\sum_{j=1}^{K} \rho_{nj}}, \\
q(\boldsymbol{\pi}) &= \mathcal{Dir}(\boldsymbol{\pi}|\boldsymbol{\delta}) \qquad \text{where} \quad \delta_k = \delta_{0_k} + \sum_{n=1}^{N} r_{nk}, \\
q(\boldsymbol{\tau}) &= \prod_{k=1}^{K} \mathcal{G}\text{amma}\left(\tau_k|\alpha_0 + \frac{D}{2}, \beta_0 + \frac{1}{2}\left\langle \mathbf{w}_k^T \mathbf{w}_k \right\rangle_{q(\mathbf{w}_k)}\right), \\
q(\mathbf{w}) &= \prod_{k=1}^{K} \mathcal{N}(\mathbf{w}_k|\mathbf{m}_k, \mathbf{S}_k), \\
q(\mathbf{Z}) &= \prod_{n=1}^{N} \prod_{i=1}^{I_n} \begin{cases} \mathcal{TN}_+\left(z_{ni}| \sum_{k=1}^{K} r_{nk} \left\langle \mathbf{w}_k^T \mathbf{x}_{ni} \right\rangle_{q(\mathbf{w}_k)}, 1\right) & \text{if } y_{ni} = 1 \\ \mathcal{TN}_-\left(z_{ni}| \sum_{k=1}^{K} r_{nk} \left\langle \mathbf{w}_k^T \mathbf{x}_{ni} \right\rangle_{q(\mathbf{w}_k)}, 1\right) & \text{if } y_{ni} = 0 \end{cases}
\end{aligned} \tag{20}$$

[3] Detailed mathematical derivations can be found in http://rpubs.com/cakapourani/vb-mixture-bpr

where

$$\ln \rho_{nk} = \langle \ln \pi_k \rangle_{q(\pi_k)} + \left\langle -\frac{1}{2} \left(\mathbf{z}_n - \mathbf{X}_n \mathbf{w}_k \right)^T \left(\mathbf{z}_n - \mathbf{X}_n \mathbf{w}_k \right) \right\rangle_{q(\mathbf{z}_n, \mathbf{w}_k)},$$

$$\boldsymbol{\lambda}_k = \mathbf{S}_k \sum_{n=1}^{N} r_{nk} \mathbf{X}_n^T \; \langle \mathbf{z}_n \rangle_{q(\mathbf{z}_n)},$$

$$\mathbf{S}_k = \left(\langle \tau_k \rangle_{q(\tau_k)} \mathbf{I} + \sum_{n=1}^{N} r_{nk} \mathbf{X}_n^T \mathbf{X}_n \right)^{-1}.$$

The variational lower bound (i.e. evidence lower bound) which we are optimising over is then given by

$$\begin{aligned}
\mathcal{L}(q) &= \sum_{\mathbf{C}} \int \int \int \int q(\mathbf{Z}, \mathbf{C}, \mathbf{w}, \boldsymbol{\pi}, \boldsymbol{\tau}) \ln \left| \frac{p(\mathbf{Y}, \mathbf{Z}, \mathbf{C}, \mathbf{w}, \boldsymbol{\pi}, \boldsymbol{\tau} | \mathbf{X})}{q(\mathbf{Z}, \mathbf{C}, \mathbf{w}, \boldsymbol{\pi}, \boldsymbol{\tau})} \right| d\mathbf{z}\, d\mathbf{w}\, d\boldsymbol{\pi}\, d\boldsymbol{\tau} \\
&= \langle \ln p(\mathbf{Y}|\mathbf{Z}) \rangle_{q(\mathbf{Z})} + \langle \ln p(\mathbf{Z}|\mathbf{C}, \mathbf{X}, \mathbf{w}) \rangle_{q(\mathbf{Z}, \mathbf{C}, \mathbf{w})} + \langle \ln p(\mathbf{C}|\boldsymbol{\pi}) \rangle_{q(\mathbf{C}, \boldsymbol{\pi})} \\
&\quad + \langle \ln p(\boldsymbol{\pi}) \rangle_{q(\boldsymbol{\pi})} + \langle \ln p(\mathbf{w}|\boldsymbol{\tau}) \rangle_{q(\mathbf{w}, \boldsymbol{\tau})} + \langle \ln p(\boldsymbol{\tau}) \rangle_{q(\boldsymbol{\tau})} \\
&\quad - \langle \ln q(\mathbf{Z}) \rangle_{q(\mathbf{Z})} - \langle \ln q(\mathbf{C}) \rangle_{q(\mathbf{C})} - \langle \ln q(\mathbf{w}) \rangle_{q(\mathbf{w})} - \langle \ln q(\boldsymbol{\pi}) \rangle_{q(\boldsymbol{\pi})} - \langle \ln q(\boldsymbol{\tau}) \rangle_{q(\boldsymbol{\tau})}.
\end{aligned} \tag{21}$$

The predictive density of a new observation $\mathbf{y}_*$ which will be associated with a latent variable $\mathbf{c}_*$, latent observation $\mathbf{z}_*$ and covariates $\mathbf{X}_*$ is given by

$$\begin{aligned}
p(\mathbf{y}_*|\mathbf{X}_*, \mathbf{Y}, \mathbf{X}) &= \sum_{c} \int \int \int \int p(\mathbf{y}_*, \mathbf{c}_*, \mathbf{z}_*, \boldsymbol{\pi}, \mathbf{w}, \boldsymbol{\tau} | \mathbf{X}_*, \mathbf{Y}, \mathbf{X})\, d\boldsymbol{\pi}\, d\boldsymbol{\tau}\, d\mathbf{w}\, d\mathbf{z}_* \\
&\simeq \sum_{k=1}^{K} \frac{\delta_k}{\hat{\delta}} \mathcal{B}ernoulli \left(y_* \middle| \Phi \left(\frac{\mathbf{X}_* \mathbf{m}_k}{\left(\mathbf{I}_n + \mathrm{diag}(\mathbf{X}_* \mathbf{S}_k \mathbf{X}_*^T) \right)^{1/2}} \right) \right)
\end{aligned} \tag{22}$$

2.2.2 Binomial observation model

Similarly to Section 2.1.2 we can transform the Binomial observations to Bernoulli observations and use the variational implementation for the Bernoulli regression model as derived above.

2.3 Model selection

One of the most appealing aspects of Bayesian inference and more specifically variational approximations within mixture models is the possibility of directly performing model selection, i.e. determining the number of clusters, within the optimisation procedure. It has been repeatedly observed (Corduneanu and Bishop, 2001) that, when fitting variationally a mixture model with a large number of components, the variational procedure will prune away components with no support in the data, hence effectively determining an appropriate number of clusters in an automatic fashion. We can gain some intuition as to why this happens in the following way. We can rewrite the $\mathcal{KL}$ divergence as

$$\begin{aligned}
\mathcal{KL}(q(\boldsymbol{\theta}) \;||\; p(\boldsymbol{\theta}|\mathbf{X})) = &\ln p(\mathbf{X}) - \langle \ln p(\mathbf{X}|\boldsymbol{\theta}) \rangle_{q(\boldsymbol{\theta})} + \\
&\mathcal{KL}(q(\boldsymbol{\theta}) \;||\; p(\boldsymbol{\theta}))
\end{aligned} \tag{23}$$

where $\ln p(\mathbf{X})$ can be ignored since is constant with respect to $q(\boldsymbol{\theta})$. To minimize this objective function the variational approximation will both try to increase the expected log likelihood of the data $\ln p(\mathbf{X}|\boldsymbol{\theta})$ while minimizing its $\mathcal{KL}$ divergence with the prior distribution p($\boldsymbol{\theta}$). Hence, using variational Bayes we have an automatic trade-off between fitting the data and model complexity (Bishop, 2006); giving the possibility to automatically determine the number of clusters without resorting to cross-validation techniques.

To provide a concrete example of model selection in the variational Bayes paradigm, we generated $\mathrm{N} = 300$ methylation genomic regions from $\mathrm{K} = 3$ clusters. Then, we set the initial number of clusters to $\mathrm{K} = 6$ and let the variational optimisation to prune away inactive clusters. Figure 2 shows the state of the variational Bayes algorithm during different iterations; and only after 15 iterations it automatically recovered the correct number of clusters. Figure 3 shows how during optimisation, the evidence lower bound increases over each iteration, including small bumps when the model discards mixture components.

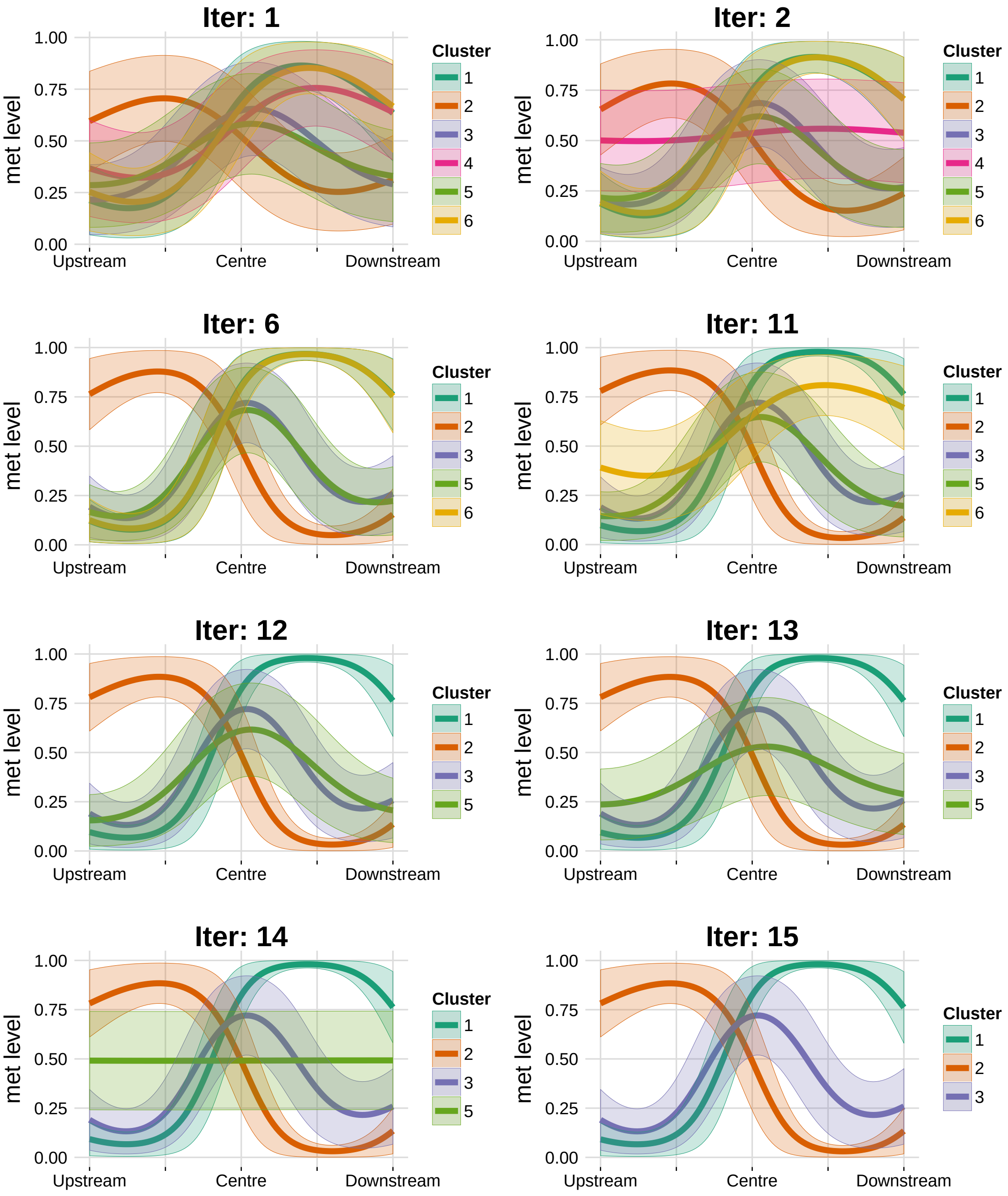

Figure 2: Variational Bayes automatically performs model selection. On synthetic data generated from three clusters, the clustering algorithm was initialized with $\mathrm{K} = 10$ clusters, and after only 15 iterations it managed to recover the correct number of clusters. Essentially, components that did not explain the data were returned back to their prior values, i.e. constant functions with 0.5 methylation level corresponding to $\mathbf{w} = \mathbf{0}$ for all basis function coefficients. These components were removed from the plots for clarity.

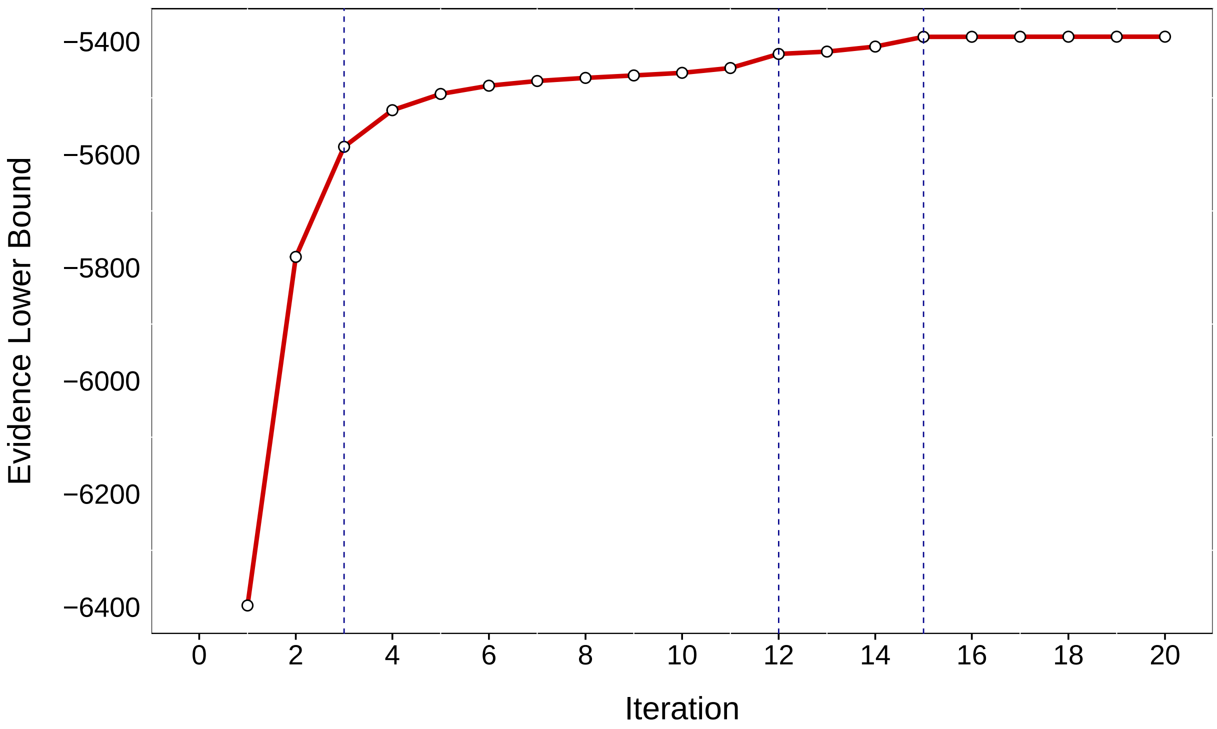

Figure 3: Evidence lower bound over the model optimization over 20 iterations for the synthetic data shown in Figure 2. Initially the model had 6 components. Each vertical blue line indicates the iteration time when a mixture component was pruned away. Note the bumps in evidence lower bound when the model discards components.

References

Albert, J. H. and Chib, S. (1993). Bayesian Analysis of Binary and Polychotomous Response Data. *Journal of the American Statistical Association*, **88**(422), 669–679.

Bishop, C. M. (2006). *Pattern recognition and machine learning*. Springer.

Blei, D. M., Kucukelbir, A., and McAuliffe, J. D. (2017). Variational Inference: A Review for Statisticians. *Journal of the American Statistical Association*, **112**(518), 859–877.

Clark, S. J., Argelaguet, R., Kapourani, C.-A., Stubbs, T. M., Lee, H. J., Krueger, F., Sanguinetti, G., Kelsey, G., Marioni, J. C., Stegle, O., and Reik, W. (2018). scNMT-seq enables joint profiling of chromatin accessibility DNA methylation and transcription in single cells. *bioRxiv*.

Corduneanu, A. and Bishop, C. M. (2001). Variational Bayesian Model Selection for Mixture Distributions. *In Artificial Intelligence and Statistics*, pages 27–34.

Ferrari, S. L. P. and Cribari-Neto, F. (2004). Beta regression for modelling rates and proportions. *Journal of Applied Statistics*, **31**(7), 799–815.

Hestenes, R. M. and Stiefel, E. (1952). Methods of Conjugate Gradients for Solving Linear Systems. *Journal of Research of the National Bureau of Standards*, **49**(6), 409–436.

Kapourani, C. A. and Sanguinetti, G. (2016). Higher order methylation features for clustering and prediction in epigenomic studies. *Bioinformatics*, **32**(17), i405–i412.

Siegmund, K. D. (2011). Statistical approaches for the analysis of DNA methylation microarray data. *Human Genetics*, **129**(6), 585–595.

Smallwood, S. a., Lee, H. J., Angermueller, C., Krueger, F., Saadeh, H., Peat, J., Andrews, S. R., Stegle, O., Reik, W., and Kelsey, G. (2014). Single-cell genome-wide bisulfite sequencing for assessing epigenetic heterogeneity. *Nature Methods*, **11**(8), 817–20.
